# The Impacts of Emotional Intelligence on Students’ Study Habits in Blended Learning Environments: The Mediating Role of Cognitive Engagement during COVID-19

**DOI:** 10.3390/bs12010014

**Published:** 2022-01-13

**Authors:** Javed Iqbal, Muhammad Zaheer Asghar, Muhammad Azeem Ashraf, Xie Yi

**Affiliations:** 1 School of Education, Guangzhou University, Guangzhou 510006, China; javed@e.gzhu.edu.cn (J.I.); soexieyi@gzhu.edu.cn (X.Y.); 2 Department of Education, University of Helsinki, 00014 Helsinki, Finland; 3 School of Doctorate, Education & ICT (e-Learning), Universitat Oberta de Catalunya, 08018 Barcelona, Spain; 4 Department of Education, University of Management and Technology, Lahore 54700, Pakistan; 5 Research Institute of Education Science, Hunan University, Changsha 410082, China; azeem20037@gmail.com

**Keywords:** emotional intelligence, cognitive engagement, study habits, blended learning, COVID-19

## Abstract

Emotional intelligence is a main area in educational psychology and a key factor in the academic life of students. It deals with deviant behavior through self-awareness and self-motivation, regulates emotional and social skills, and converts emotional energy into positive energy. This study examined direct and indirect relationships between emotional intelligence and study habits in blended learning environments. Blended learning is conceptualized as a hybrid learning approach that combines online learning opportunities and the traditional classroom approach. Furthermore, the study explored the mediating role of cognitive engagement in the relationship between emotional intelligence and study habits. We used 26 items in a paper-based questionnaire in a quantitative study to collect data on emotional intelligence, cognitive engagement and study habits from health sciences students (N = 338) enrolled in blended learning courses in universities in the Hunan province of China. Emotional intelligence included self-awareness, self-motivation, and the regulation of emotion; social skills were also examined. A partial least squares structural-equation modeling approach was applied through SmartPLS software to explore the relationships. The results indicate that self-awareness and self-motivation have direct, significant, and positive connections with study habits. Similarly, the results indicate that all four dimensions of emotional intelligence (self-awareness, self-motivation, emotion regulation and social skills) had indirect, significant, and positive relationships with study habits using cognitive engagement as a mediator variable. It was concluded that students face higher-than-usual challenges in building study habits in blended learning environments during the COVID-19 pandemic, and that emotional intelligence helps them to develop their study habits to greater effect. Similarly, it was concluded that cognitive engagement strengthens the connection between emotional intelligence and study habits. Therefore, it is recommended that universities take specific measures to enhance students’ emotional intelligence and cognitive engagement, which will ultimately improve their study habits. Moreover, valuable and practical implications for teachers, practitioners, and university management were also discussed in the study.

## 1. Introduction

Emotional intelligence works as an essential predictor of student learning and cognitive health [1]. It also provides timely psychological support to cognitive engagement and study habits in the COVID-19 era. Students and teachers have managed the learning process during the pandemic through their emotional intelligence and cognitive engagement in blended learning environments [2]. Blended learning is a hybrid learning approach that combines online learning opportunities and the traditional classroom approach. Higher education institutions have managed the issue of campus closure by shifting classes from face-to-face to online [3]. The situation may affect students’ study habits. Such circumstances provoked the authors to explore the connection between emotional intelligence, cognitive engagement, and study habits in blended learning environments.

Emotional intelligence (EI) is a key area of psychology and an important factor in the academic life of students [4]. It deals with deviant behavior through self-awareness and self-motivation, regulates emotional and social skills, and converts emotional energy into positive energy [5]. Researchers have examined the connection between emotional intelligence with other variables, such as stress management, exam anxiety, and problem-solving abilities. Furthermore, some have posited that students with high emotional intelligence perform well in dealing with academic challenges [4]. Researchers Parker, et al. [6] concluded that EI is a positive predictor in learning. In this study, the emotional intelligence construct was explored alongside study habits (SH) in the context of health education in China.

Study habits provide academic stability and are necessary for academic success [7]. These habits can be improved through various practices and training [8]. Various studies have discussed the factors that affect study habits [9]. Universities are constantly taking measures to improve study habits [10]. Emotional intelligence is considered an important element in developing study habits [11].

Cognitive engagement is an essential predictor of learning in class. In cognitive engagement, students learn how previous knowledge can be utilized to relate to real-life problems and learn how to handle hands-on tasks [12]. Cognitive engagement effectively works with intellectual factors such as self-regulation and desired learning goals [13]. Many studies have explored cognitive engagement with different constructs in the field of educational psychology. One study [13] concluded that cognitive engagement is positively connected with academic motivation. Cognitive engagement is a critical factor that needs to be explored in a blended learning context in China. This study explores the mediating role of cognitive engagement in the association between emotional intelligence and study habits.

Despite the previous researchers’ strong focus and interest, some gaps remain in developing interactions among emotional intelligence, cognitive engagement, and study habits. First, most of their studies concentrated on the exercise of emotional intelligence, cognitive engagement, and study habits in advanced countries such as Australia, certain European nations, the United States, Singapore, Korea, Japan, Russia, and New Zealand [14,15,16]. Only a few studies have been conducted in emerging nations [17]. Moreover, the higher education sector in the COVID-19 era focuses on blended learning mode classes and creates psychological issues owing to unpredictable situations. Therefore, it was important to explore emotional intelligence, cognitive engagement, and study habits in these circumstances [18]. Third, few studies have explored the direct connections between EI and study habits. Given this research gap, this study investigates the influence of emotional intelligence on study habits. The next section explores the mediating effect of cognitive engagement. To offer a wide range of perspectives within the phenomenon, it also considers various variables highlighted in the literature as illuminating the connection between emotional intelligence and study habits, namely cognitive engagement. Therefore, the research questions that guide this study are as follows:

**Research** **Question** **1.** *How do emotional intelligence and cognitive engagement influence study habits?*

**Research** **Question** **2.** *How does cognitive engagement mediate the relationship between emotional intelligence and study habits?*

This study used a quantitative approach to examine China, focused on 338 health sciences students, and applied SEM (structural equation modeling) to test a combination of research hypotheses. This study drew on the literature in terms of theoretical, methodological, empirical, and practical implications as follows: first, it augments theory in the literature by finding the effect of emotional intelligence on the study habits of higher education students with the mediating role of cognitive engagement. It augments knowledge via the sociological perspective of COVID-19, pedagogically blended learning environments, and, geographically, from medical universities located in China. Second, the theoretical addition was escalated through partial least square equation modeling of exogenous, mediating, and endogenous constructs from the interdisciplinary perspectives of the area of psychology, instructional design, and pedagogy. Third, this study provides robust statistical evidence from an adequate number of samples selected from medical students. Finally, the study has practical implications for higher education policy makers in the field of medical education, curriculum development, and instructional design during emergencies such as the COVID-19 pandemic. This study will be helpful for medical education stakeholders to understand the psychological, sociological, and instructional environmental aspects of students as they continue their education in blended learning. 

The authors organized the rest of the study as follows: Part two contains the literature review and conceptual framework with hypotheses formulation; Part 3 describes the research methodology; Part 4 comprises data analysis and interpretation; and Part 5 discusses the results. Parts 6 and 7 supply the conclusions and implications, respectively. 

## 2. Literature Review

### 2.1. Research Framework

Emotional intelligence plays an essential role in academic success, particularly during the pandemic (COVID-19). Most studies have discussed the theoretical and practical issues regarding student emotional and mental health. As Cleary et al. [19] explained, emotional intelligence guides the cognition drive in student academic life. Studies on emotional intelligence have received much attention over the last two decades in the Chinese context [20,21,22,23,24]. Moreover, recent studies have concentrated on the influence of emotional intelligence on behaviors and the role of training in social skills challenges in the twenty-first century [25]. Similarly, Chen and Guo [26] discussed the role of emotional intelligence in school education. Furthermore, emotional intelligence has become a popular concept among scholars in China, many of whom are focusing on the role of emotional intelligence to enhance academic performance in Chinese higher education [27]. Another study discussed the impact of emotional intelligence on teacher–student relationships and social problem-solving skills [28]. Moreover, blended learning is considered a mix of different learning approaches. This study has operationalized blended learning as mix of face-to-face and online learning. Both online learning and face-to-face learning approaches have their pros and cons in terms of the COVID-19 pandemic. For example, when face-to-face learning was ceased, online learning provided an alternative. However, it often inhibits student engagement and socialization. The blended learning approach compensates for the drawbacks of both face-to-face and online learning. The positive aspects of both approaches—i.e., the socialization and engagement aspects of the face-to-face approach and the accessibility benefit of the online approach—helped students to continue education during emergency [29].

**Figure 1:**
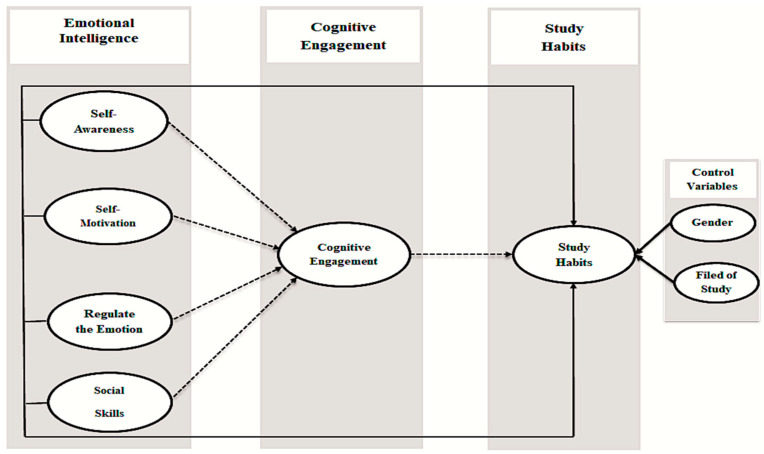
Research framework.

In this study, the proposed research framework demonstrates the influence of emotional intelligence on study habits through cognitive engagement. Emotional intelligence and cognitive engagement are of great concern for researchers examining study habits among students. Emotional intelligence is a positive predictor of cognitive engagement, and study habits have been claimed by various scholars [11,30,31] in the field of educational psychology. Emotional intelligence factors such as self-awareness, self-motivation, the regulation of emotion, and social skills were also discussed in various studies [32,33,34]. The theory of student involvement provides the basis for designing the research model [35]. In the academic environment, emotional intelligence has been shown to have a positive impact on cognitive engagement that may be helpful in improving study habits. The authors hypothesized the cognitive engagement connections between emotional intelligence and study habits, and sought to explore the mediated influence of cognitive engagement. The study analyzes these relationships empirically and sheds light on the effects of EI on study habits through cognitive engagement in an emerging country. The research also correlates previous studies by demonstrating the impacts of emotional intelligence in enhancing the cognitive engagement that leads to study habits. Based on these discussions and the reviewed literature, these relationships are shown in Figure 1.

### 2.2. Hypotheses Formation

#### 2.2.1. Emotional Intelligence and Study Habits

**Figure 2:**
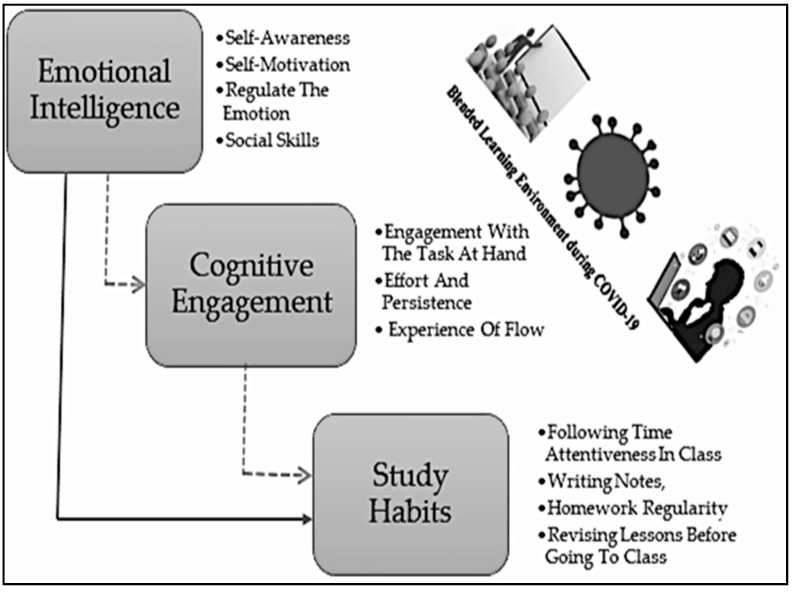
Literature flow.

Emotional intelligence is an important factor in helping students perceive emotions properly and in understanding the emotions of themselves and others. The emotional intelligence dimension regulates emotions through various strategies, and alleviates stressful situations through emotional intelligence knowledge [24,36]. In a difficult situation, a person with a good level of emotional intelligence can handle others well. However, the individual with low emotional intelligence has to face vulnerable situations such as anxiety, stress, and burnout [37]. Therefore, self-awareness, self-motivation, regulation of emotion, and social skills may help individuals in handling difficult situations [32,38]. Self-awareness is the ability to identify one’s own and others’ emotions. Self-motivation assists in the ability to complete tasks through inspiration to accomplish objectives under challenging situations. The regulation of emotions is defined as redirecting emotions and predicting repercussions before acting. Social skills are attributes that maintain interaction with others either through prompted responses or motivation [34]. Thus, this study explored the influence of emotional intelligence on study habits (Figure 2).

Study habits are general abilities that help students enhance their academic performance [39]. Study habits are defined as the normal tendencies and practices that one practices while receiving information through learning. Moreover, study habits are conceptualized as how students adapt through the learning process; for example, following time, attentiveness in class, writing notes, doing homework regularly, and revising lessons before going to class [39]. Furthermore, it has been discussed that poor study habits have adverse effects on learning and academic performance [40]. A number of studies have explored the effect of various factors on study habits such as the demographical characteristics of gender, age and race. Several psychological characteristics were also explored, such as self-efficacy, academic motivation, optimism, time management skills, and student performance [39]. Research on study habits such as learning strategies, concentration in class, being on time, taking solid notes, and accomplishing homework were also explored. 

Various researchers and academicians have highlighted the positive associations of emotional intelligence and dimensions, e.g., self-awareness, self-motivation, regulation of emotion, and social skills with study habits [11]. Multiple studies have also been designed on the specific topic of emotional intelligence dimensions and their effects on study habits; these also support the existence of positive relationships [30]. Student involvement theory also supports the notion that psychological well-being is important in terms of study habits, and that emotional intelligence is a key predictor in the teaching–learning process [27,31,35]. Furthermore, student involvement theory explains that emotional intelligence and cognitive engagement may be considered the key elements for study habits [41,42]. The discussion has helped researchers to understand the relationships between emotional intelligence and study habits. As such, the positive association between emotional intelligence and study habits was assumed in the following hypothesis:

**Hypothesis** **1.1.** *Self-awareness has a positive influence on study habits.*

**Hypothesis** **1.2.** *Self-motivation has a positive influence on study habits.*

**Hypothesis** **1.3.** *Regulation of emotion has a positive influence on study habits.*

**Hypothesis** **1.4.** *Social skills have a positive influence on study habits.*

#### 2.2.2. Emotional Intelligence and Cognitive Engagement

Emotional intelligence dimensions such as self-awareness, self-motivation, regulation of emotion, and social skills have positive effects on cognitive engagement [41]. Similarly, the theory of student involvement provides insights, as emotional intelligence has a positive role in developing students’ attitude towards learning in higher education [35,43]. Chong, Liem, Huan, Kit and Ang [13], and some researchers have investigated the role of emotional intelligence and self-efficacy in improving cognitive engagement. In another study, emotional intelligence was identified as the positive predictor of cognitive engagement among higher education students [13]. It has also been noted that emotional intelligence deals with the multidimensional nature of cognitive engagement and recognizes its role in improving academic success [44]. 

Cognitive engagement is the ability of students to make an effort to comprehend their own learning and to relate learning content to real-life problems over a long period of time. Students spend their energy planning for learning with motivation, willingness, and commitment [45,46]. It has traditionally been operationalized by assessing students’ home assignments, extra-curricular activity participation, and their interaction with peers and teachers in either online or face-to-face learning [45]. It includes the autonomy in activity or task, which largely determines where and how students engage cognitively in their tasks [47]. It includes factors such as peer discussions, searching online information on the internet, attending lectures, independent work, and discussion [48]. In this study, we adopted the indicators for measuring cognitive engagement from the work of Iqbal, Qureshi and Asghar [45]. Student cognitive engagement can be measured in the context of students’ attention in learning activities, such as a shift from misconception to learning, social interaction with mentors, writing notes, and processing new information and knowledge [49]. Similarly, this study has utilized student cognitive engagement in terms of three main elements: (1) engagement with the task at hand, (2) effort and persistence, and (3) experience of flow [50]. Cognitive engagement was assessed with two additional psychological factors: emotional intelligence and study habits. In line with the discussion, emotional intelligence might have a positive relationship with cognitive engagement. Thus, the following hypotheses were made to measure relationships:

**Hypothesis** **2.1.** *Self-awareness has a positive influence on cognitive engagement.*

**Hypothesis** **2.2.** *Self-motivation has a positive influence on cognitive engagement.*

**Hypothesis** **2.3.** *Regulation of emotion has a positive influence on cognitive engagement.*

**Hypothesis** **2.4.** *Social skills have a positive influence on cognitive engagement.*

#### 2.2.3. Cognitive Engagement and Study Habits

Student engagement assumes a central role in developing study habits among students, and cognitive engagement is a dimension of student engagement. The result of one study [30] revealed that cognitive engagement has a positive role in developing study habits. Multiple factors were discussed, including student engagement and the positive role it plays in developing study habits and cognitive skills [51]. Researchers Bresó et al. [52] noted that the cognitive engagement element has a very effective role in increasing study belief and school engagement, both of which may be helpful in learning. Hence, a positive relationship of cognitive engagement with study habits is assumed in the following hypothesis:

**Hypothesis** **3.** *Cognitive engagement has a positive influence on study skills.*

#### 2.2.4. Mediating Effects of Cognitive Engagement

Previous studies have indicated that cognitive engagement is a positive predictor of learning outcomes [53]. Most studies have indicated that emotional intelligence and cognitive engagement determine student learning outcomes [54]. One particular study explored the relationship of social intelligence and study habits [11]. The theory of student involvement explains the indirect relationship of emotional intelligence with study habits, along with cognitive engagement as an supplemental variable [35]. The literature demonstrates the gaps in the notion that cognitive engagement functions as a key mediator between emotional intelligence and study habits. Hence, the following hypotheses were put forward to measure these relationships:

**Hypothesis** **4.1.** *Cognitive engagement mediates the relationship between self-awareness and study habits.*

**Hypothesis** **4.2.** *Cognitive engagement mediates the relationship between self-motivation and study habits.*

**Hypothesis** **4.3.** *Cognitive engagement mediates the relationship between regulation of emotion and study habits.*

**Hypothesis** **4.4.** *Cognitive engagement mediates the relationship between social skills and study habits.*

## 3. Research Methods

We selected the higher education sector in an emerging part of China as the site of our research for the following reasons. The majority of previous studies related to the area were executed in developed countries. There is relatively less research in emerging countries with a diverse approach to study skills in blended learning courses. Currently, China is establishing a blended learning setup in its higher education system, which creates an impetus for the research. COVID-19 has affected student study habits across the world, as well as across Chinese universities. Therefore, it was important to investigate how emotional intelligence and cognitive engagement influence study habits among health sciences students during COVID-19. Furthermore, the site was accessible to the authors. We used a cross-sectional survey approach for the study. A survey questionnaire was used to collect data for the study. The questionnaire was divided into three sections: (1) orientation (research purpose, confidentiality, privacy, and anonymity), (2) demographic information (gender, background, age, and field of study), (3) and constructs of related items (emotional intelligence, cognitive engagement, and study habits).

### 3.1. Questionnaire Design 

We used the survey questionnaire technique for the research. This technique is used for a wide range of data collection in empirical studies [55,56]. The questionnaire comprised twenty-six statements. Each of the statements offered respondents choices on a 7-point Likert scale. The items were adapted and modified from the work of Iqbal and Qureshi [34], Iqbal, Qureshi and Asghar [45], and a literature review [57,58,59]. Emotional intelligence, along with its domains such as self-awareness (3-items), self-motivation (3-items), regulation of emotion (4-items), and social skills (3-items), was assessed through 13 items. Cognitive engagement was measured on 6 items, while the study habits contained 7 items. The original questionnaire was in English, and we also conducted the survey in English. A pilot study was run with 30 participants having similar profiles, and the final sample was performed to measure the reliability of the questionnaire. Based on participants’ feedback, we made corrections in terms of context adaptation, face validity appropriation, and academic writing corrections, and it was assured that all items used in the final questionnaire would be well understood by the participants; the participants filled out the questionnaires successfully. 

### 3.2. Measures 

#### 3.2.1. Emotional Intelligence

##### Self-Awareness

The three items for self-awareness were adapted from the work of Iqbal and Qureshi [34]. Three items were used for the collection of responses on a 7-point Likert scale (“strongly disagree” to “strongly agree”). Examples of items include: “I can identify my emotions in different situations” and “I know my moods are easily affected by external events”.

##### Self-Motivation

The three items for self-motivation were adapted from the work of Iqbal and Qureshi [34]. Three items were used for the collection of responses on a 7-point Likert scale (“strongly disagree” to “strongly agree”). Examples of items include: “I accept responsibility for my reactions” and “I learn to do better next time”.

##### Regulation of Emotion

The four items for regulation of emotion were adapted from the work of Iqbal and Qureshi [34]. Three items were used for the collection of responses on a 7-point Likert scale (“strongly disagree” to “strongly agree”). Examples of items include: “I can talk to someone if I am very upset” and “I concentrate on a pleasant activity when I am feeling low”.

##### Social Skills

The three items for social skills were adapted from the work of Iqbal and Qureshi [34]. Three items were used for the collection of responses on 7-point Likert scale (“strongly disagree” to “strongly agree”). Examples of items include: “I find it easy to share my feelings with others” and “It is easy for me to make friends”.

##### Cognitive Engagement

The six items for cognitive engagement were adapted from the work of Iqbal, Qureshi and Asghar [45]. Three items were used for the collection of responses on a 7-point Likert scale (“strongly disagree” to “strongly agree”). Examples of items include: “I can relate the lessons learned in the classroom with a solution to the real-life problem,” and “I engage myself in frequent debates and discussions about problems that arise in the class during a lesson.

##### Study Habits

The seven items for study habits were adapted from the work of Iqbal, Qureshi and Asghar [45], Ayodele and Adebiyi [57] and Iqbal et al. [58]. Three items were used for the collection of responses on a 7-point Likert scale (“strongly disagree” to “strongly agree”). Examples of items include: “I do my assignments regularly” and “I attend classes regularly”.

## 4. Data Collocation

We collected data from the six universities of Hunan province that offer blended learning courses in health sciences through the stratified random sampling technique. To ensure participants’ privacy and confidentiality, the university names were listed as university A, B, C, D, E, and F. We selected students who had completed three years of study because they were experienced in both face-to-face and online classes; students with no experience of online or face-to-face classes were excluded. They completed the questionnaire with paper and pencil. Proper campus environments and time were provided to them to fill the questionnaires. We circulated 450 copies of the questionnaire among students and received 352 questionnaires in return, 14 of which were rejected because they were incomplete and not appropriate for data analysis. The final sample comprised 338 responses.

## 5. Data Analysis Procedures

We adopted the SmartPLS (version 3.3.3) and SPSS software for data analysis. First, we analyzed measurement modeling such as factor loading, Cronbach’s alpha, rho_A, and composite reliability for reliability measures. Second, the convergent and discriminant validity were also ensured. At the third level, we analyzed demographic variables through SPSS. Before structural equation modeling, we also analyzed model fit, collinearity, and R-square. Fourth, the descriptive analysis was given. Fifth, we described the results of structural modeling. Similar analysis approaches were used by different studies.

### 5.1. Measurement Model

The CFA (confirmatory factor analysis) was used to measure the validity and reliability of the scales [60] through SmartPLS (version 3.3.3). SmartPLS is recommended as it is the least sensitive to sample size and more statistically efficient than other statistical packages that are used in covariance-based structural equation modeling [61]. We used Covariance-based SEM for three reasons. First, it helps in theory exploration by measuring the cause and effect between exogenous and endogenous constructs. Second, CB-SEM is useful for complex multivariate analysis. Third, unlike the AMOS-based SEM approach, it does not require strict assumptions of data normality and sample size [62]. The option of bootstrapping enabled the CB-SEM to run data analysis by standardizing the indicators [63]. Our study also aimed to discover the cause and effect between the exogenous construct of emotional intelligence and study habits. Moreover, it introduces complex multivariate analysis with the mediation of cognitive engagement. Therefore, CB-SEM was considered the best option for data analysis in our study.

We applied structural equation modeling to test the associations of variables used in the research model [64]. We measured the direct and indirect relationships among emotional intelligence (self-awareness, self-motivation, regulation of emotion, and social skills) and study habits. Cognitive engagement was used as a mediator variable between emotional intelligence subscales and study habits. We ensured the validity and reliability of each construct before the start of final data analysis. The reliability and validity of each construct were ensured through a measure modeling analysis approach prior to SEM analysis. Factor loading, Cronbach’s alpha, rho_A, and composite reliability and AVE (average variance extracted) techniques were used [65,66]. The threshold value of factor loading for each item was higher than 0.60 [66]. The threshold values of Cronbach’s alpha, rho_A, and composite reliability were also higher than 0.70 [67]. Moreover, convergent validity was tested through AVE. The AVE value for all constructs should be above 0.5 [61,67]. Table 1 shows that the factor loading figures are above the threshold value of 0.6. Cronbach’s alpha, rho_A, and composite reliability values are also above 0.70. The AVE value is above 0.5, so it is concluded that the scale used in the study was reliable and valid (see Table 1).

Discriminant validity can be measured through the heterotrait:monotrait (HTMT) ratio [61]. The researchers [61] used the HTMT approach for measuring the discriminant validity of reflective scales used in the research model. The HTMT approach means that the mean score value of the item correlations among constructs relate to the geometric mean of the average relationships among evaluated items with their relevant construct [61]. The approach is more valid and reliable in SEM analysis as compared with other approaches such as Fonrell and Larcker’s criterion [68] approach, which has been criticized by researchers [69]. Fornell and Larcker’s approach is not strong enough to produce efficient results as compared with HTMT Henseler, Dijkstra, Sarstedt, Ringle, Diamantopoulos, Straub, Ketchen Jr, Hair, Hult and Calantone [61], and suggests less than the 0.90 threshold value which exists for HTMT [70]. The value above 0.90 for HTMT means that discriminant validity issues exist [71]. The results in Table 2 reveal that the HTMT values of each construct are less than 0.90. Thus, the scale fulfilled requirements regarding discriminant validity (see Table 2). 

### 5.2. Demographic Analysis

There were 338 usable copies of the questionnaire returned. We used descriptive statistics to analyze the demographic profiles of the participants. Among the participants, 54.6% were males, while females comprised 45.4%; rural participants numbered 40.7%, and urban 59.3%; in terms of age, less than 22 comprised 75.3% of the participants, 22–30 comprised 24.7%; in terms of medical imaging, students in ultrasonography numbered 46.1%, nutrition sciences 23.2%, and physiotherapy 30.7%. Details of participants’ demographic data are shown in Table 3. 

The structural equation modeling technique generally assured the elimination of the problem of collinearity among variables through the Variance Inflation Factor (VIF). The threshold value for VIF is less than 5. A VIF value above 5 indicates the presence of a collinearity problem between variables [67]. In the study, the value of VIF is less than 5, which is between 1.320 and 1.737, and indicates no collinearity problem among the variables. 

We used SRMR, NFI and RMS_theta in SmartPLS indicators to fit the model. The threshold value of SRMR is from 0 to 1, and less than 0.80 is considered ideal for a good fit [72]. The NFI range is between 0 and 1, and greater than 0.90 is suitable for the appropriateness of the overall model [72,73]. The RMS_theta is the most suitable indicator for assessing reflective measurement models, and the threshold value for good model fit is less than 0.12 [61]. In this study, the value of SRMR is 0.073, which is less than 0.80. The value of NFI is 0.873, and it is less than 0.9. It shows little difference [74]. The RMS_theta value is 0.13. It is not much higher than the ideal value, and it is also appropriated. Therefore, it is concluded that the model of the study is reasonably well-fitted in general. The analysis of collinearity and model fits are provided in Table 4.

In SEM analysis, we assessed the explanatory power of the output model with the range of *R*^2^ between 0 and 1. The threshold values of *R*^2^ are up to 0.25, 0.50, and 0.750, which are considered as weak, moderate, and strong explanatory power, respectively [67]. Table 5 indicates the explanatory power of cognitive engagement and study habits, which are 0.444 and 0.407, respectively. Both dimensions have a moderate degree of explanatory power. Therefore, the model in the study suggests that the latent variables are appropriate at the degree of explanatory power. 

#### Descriptive Analysis

Table 6 explains the descriptive statistics of the survey respondents. The 7-point Likert scale was applied to record the responses. The 338 responses were usable. The range of mean values for all responses falls between 4.650 and 5.288. The range of standard deviation falls between 1.141 and 1.324. Details concerning the descriptive statistics of survey respondents are revealed in Table 6. 

### 5.3. Structural Equation Modeling

Structural equation modeling was applied through SmartPLS with the bootstrapping technique (5000). In this study, we used the technique to measure path estimates, *p*-values, *t*-values, and confidence intervals [64]. Direct and indirect relationships were measured among the constructs used in the research model. The results of the analysis show that self-awareness shows a positive and significant relationship with study habits (β = 0.121, *p* < 0.05), and approves hypothesis H1.1. Self-motivation shows a significant and positive correlation with study habits (β = 0.123, *p* < 0.05), which approves hypothesis H1.2. On the other hand, regulation of emotion does not show a positive and significant connection with study habits (β = 0.094, *p* > 0.05), which does not approve hypothesis H1.3. Correspondingly, the results indicate that social skills do not have a positive and significant association with study habits (β = 0.078, *p* > 0.05), which does not support hypothesis H1.4. Moreover, the results indicate that self-awareness has a positive and significant connection with cognitive engagement (β = 0.164, *p* < 0.05), which approves hypothesis H2.1. Self-motivation has a positive and significant connection with cognitive engagement (β = 0.134, *p* < 0.05), which supports hypothesis H2.2. Emotional regulation has a positive and significant association with cognitive engagement (β = 0.330, *p* < 0.05), which approves hypothesis H2.3. Additionally, social skills have a positive and significant connection with cognitive engagement (β = 0.252, *p* < 0.05), and hypothesis H2.4 is accepted. Finally, cognitive engagement has a positive and significant association with study habits (β = 0.370, *p* < 0.05), which approves hypothesis H3. We also measured two control variables, gender and field of study. Of these, only gender indicated a significant influence on study habits (β = 0.117, *p* < 0.05). Table 7 supply more details.

**Figure 3:**
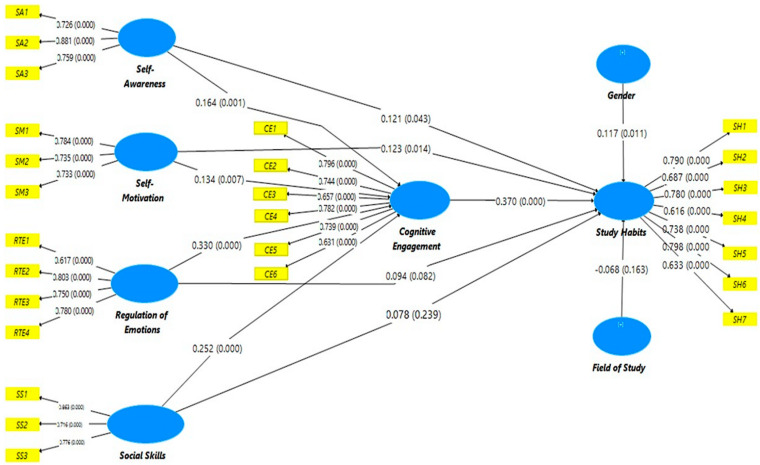
A path analysis model of emotional intelligence and study habits through cognitive engagement.

In the study, we measured the indirect relationship of emotional intelligence (i.e., self-awareness, self-motivation, regulation of emotion, and social skills) with study habits, while cognitive engagement was used as a mediator variable. PLS-SEM considers the mean values in path analysis, such as the regression coefficient. In Table 8, the results indicate that cognitive engagement mediated the association between self-awareness and study habits (β = 0.061, *p* < 0.05); therefore, hypothesis H4.1 is accepted. Cognitive engagement mediated the relationship between self-motivation and study habits (β = 0.049, *p* < 0.05), and approves hypothesis H4.2. Cognitive engagement mediated the connection between regulation of emotion and study habits (β = 0.122, *p* < 0.05), and approves hypothesis H4.3. Lastly, cognitive engagement mediated the connection between social skills and study habits (β = 0.093, *p* < 0.05). Thus, hypothesis H4.4 is approved. The results indicate that all the hypotheses listed in Table 8 are approved. Figure 3 also shows the path coefficients of the model.

## 6. Discussion

This study explored the connections between emotional intelligence and study habits through cognitive engagement among students in blended learning environments during COVID-19. The study made an effort to determine meaningful results based on the synthesized research model. Similar studies have been conducted on the same issue in advanced countries [16,31,75], and only limited studies have been performed in emerging countries such as China [41]. Furthermore, the studies that have been conducted in emerging countries reveal results only in pre-COVID-19 situations. As such, the authors claim that the present study is the first to examine the influence of emotional intelligence on the study habits of students studying in blended learning environments in Chinese universities, specifically as it focuses on cognitive engagement as a mediator variable. 

First, the study measured the association between emotional intelligence and study habits. The results indicate that self-awareness and self-management positively and significantly influence study habits, which approves hypotheses H1.1 to H1.2. Prior studies also confirmed that self-awareness and self-motivation have a positive connection with study habits [76,77]. The relationship between emotional intelligence and study habits has been discussed in previous studies, and their results confirmed that emotional intelligence promotes a positive relationship with study habits [78,79]. However, in the research, the associations of emotional intelligence dimensions, regulation of emotion, and social skills were found to be positive and insignificant with regard to study habits. A plausible reason for these results could be that universities did not fully prepare their students to develop emotional intelligence in the area of regulation of emotion and social skills. There could be other reasons for these results, such as the students’ weak social skills and poor regulation of emotion owing to fewer face-to-face interactions in blended learning environments during the COVID-19 pandemic. 

Second, the results show that self-awareness, self-motivation, regulation of emotion, and social skills have a positive influence on cognitive engagement, which approved hypotheses H2.1 to H2.4. Earlier studies also confirmed the connection between emotional intelligence dimensions and cognitive engagement [80]. The researchers in Perera and DiGiacomo [81] posited that emotional intelligence remains a positive predictor of cognitive engagement. Therefore, it was concluded that emotional intelligence dimensions are important factors which provide valuable support to cognitive engagement. Another factor which might account for increased cognitive engagement is that blended learning environments provide students with opportunities to learn virtually and face-to-face more easily, managing their engagement level more effectively during COVID-19. Their non-academic activities were limited, and as such they may have been more focused on emotional well-being, which ultimately increased their cognitive engagement in blended environments. 

Third, the results of the study reveal the direct influence of cognitive engagement on study habits, which approved hypothesis H3. Previous research also confirms that cognitive engagement has a positive effect on study habits [42]. Researchers Bilge, Tuzgol Dost and Cetin [30] examined the relationship between student engagement and study habits among students; the results of the study found that student engagement has a positive relationship with study habits. Thus, it was concluded that cognitive engagement has a positive role in developing study habits. There could be other reasons that enhance the relationship, such as efficient internet access, smart phone access, efficient teachers, and more developed emotional intelligence and cognitive engagement in blended learning environments, such as during the COVID-19 pandemic. 

Fourth, the mediated relationship also indicated significant results, which constitutes an original contribution to the research in the context of China. Cognitive engagement mediated the connection between emotional intelligence dimensions (self-awareness, self-motivation, regulation of emotion, and social skills) and study habits, which approved our intuitions in hypotheses H4.1 to H4.4. The results of a prior study supported our results that cognitive engagement mediated the relationship between EI and study habits [82]. Researchers Pietarinen, Soini and Pyhältö [54] have suggested that the relationship between cognitive engagement and study habits, along with emotional intelligence and academic outcomes, needs to be examined further. Thus, it was deduced and proved that cognitive engagement and emotional intelligence (self-awareness, self-motivation, regulation of emotion, and social skills) are the main predictors of study habits in blended learning environments in China during the COVID-19 pandemic. 

## 7. Conclusions

The synthesized research model was developed based on the previous literature [27,30,54]. The results reveal direct associations between emotional intelligence, cognitive engagement and study skills in an emerging country, China, during COVID-19. The study indicates that certain emotional intelligence dimensions (self-awareness and self-motivation) have a direct, positive, and significant impact on study habits. The other two dimensions of emotional intelligence (regulation of emotions and social skills) have an insignificant positive influence on study habits. The results also reveal that emotional intelligence dimensions such as self-awareness, self-motivation, regulation of emotion, and social skills have a positive and significant effect on cognitive engagement in blended learning environments. Additionally, the results indicate that cognitive engagement has a positive role in improving study habits. Moreover, our results indicate that all four emotional intelligence dimensions, including self-awareness, self-motivation, regulation of emotion, and social skills, have an indirect, positive, and significant influence on study habits through cognitive engagement in COVID-19. 

The results could be interpreted as follows: in the higher education sector, self-awareness and self-motivation are positive predictors of study habits among students. On the other hand, it was concluded that emotional intelligence dimensions such as regulation of emotion and social skills were not associated with study habits. Similarly, emotional intelligence (self-awareness, self-motivation, regulation of emotion, and social skills) is considered a critical predictor for developing cognitive engagement. Furthermore, cognitive engagement has a very significant role in developing and improving study habits. Likewise, it was concluded that cognitive engagement, along with emotional intelligence, improved study habits. It is very important to understand that students who are intelligent and disciplined to begin with will maintain good study habits in any conditions. Students with advanced levels of emotional intelligence likely have a high level of cognitive engagement in any conditions. These conclusions provide the understanding that people who work in higher education can increase the level of emotional intelligence of their students through organizing cohort courses, boot camps, seminars, and workshops, and also by offering integrated courses to their students. Specifically, emotional intelligence, particularly in regard to regulation of emotion and social skills, requires special care for students. Emotional intelligence not only enhances cognitive engagement but also improves study habits directly and indirectly. 

### 7.1. Implications

Emotional intelligence and cognitive engagement are significant factors in developing study habits among students. The COVID-19 pandemic created a disrupted environment, and universities shifted their teaching to blended learning modes to cope with the pandemic’s challenges. It is expected that the universities will pay attention to factors that influence study habits. Universities in China should pay more attention to students’ emotional intelligence and cognitive engagement as a means of promoting study habits in blended learning environments. Emotional intelligence and cognitive engagement could be effective ways to cope with pandemic conditions in emerging countries such as China. Universities should offer emotional intelligence courses and increase students’ cognitive engagement levels so that students might develop better study habits.

This study suggests some practical implications to improve study habits among students in blended learning environments. First, universities should introduce an integrated curriculum of emotional intelligence and cognitive engagement to develop study habits among students. Second, it is very important for teachers to make sure one’s teaching is effective in both delivering knowledge and maintaining focus on the students themselves. Teachers should identify which students have low emotional intelligence and need emotional support for cognitive engagement so that those students can improve their study habits. Third, university management should offer psychological counseling to help teachers identify emotionally weak students. Fourth, curriculum designers should promote content in emotional intelligence and cognitive engagement so that these two factors may assist students in developing their study habits. 

### 7.2. Limitations and Future Research 

Although our study has several strengths, it also encountered limitations. The participants belonged only to one country, which may have led to cultural bias and affected the generalizability of the results. To address this deficiency, studies in other cultural situations within China—as well as in other countries—are needed. Furthermore, the data were collected only from health sciences students; students from the social sciences, business sciences, and natural sciences were not included in the study. Future research should include students from social science, business sciences, and natural sciences. This study investigated the relationships among emotional intelligence, cognitive engagement and students’ study habits; it would be a valuable addition to the present work for researchers to investigate the relationship between emotional intelligence and self-growth, using social skills as a mediating variable. Our theoretical model covered three main latent variables, emotional intelligence, cognitive engagement and study habits, along with two control variables, gender and age. Future research may supplement this background information with additional control variables such as internet access and speed, with computer devices as mediating variables.

## Figures and Tables

**Table 1:** Reliability and convergent validity.

Dimensions of Constructs	Loading	œ	rho_A	CR	AVE
Self-Awareness (SA)		0.700	0.719	0.833	0.627
SA1	0.726				
SA2	0.881				
SA3	0.759				
Self-Motivation (SM)		0.714	0.717	0.795	0.564
SM1	0.784				
SM2	0.735				
SM3	0.733				
Regulation of Emotion (RE)		0.731	0.755	0.828	0.549
RE1	0.617				
RE2	0.803				
RE3	0.750				
RE4	0.780				
Social Skills (SS)		0.701	0.715	0.829	0.620
SS1	0.863				
SS2	0.716				
SS3	0.776				
Cognitive Engagement (CE)		0.820	0.831	0.87	0.529
CE1	0.796				
CE2	0.744				
CE3	0.657				
CE4	0.782				
CE5	0.739				
CE6	0.631				
Study Habits		0.846	0.855	0.884	0.523
SH1	0.791				
SH2	0.687				
SH3	0.680				
SH4	0.617				
SH5	0.738				
SH6	0.798				
SH7	0.633				

**Table 2:** Discriminant validity (HTMT).

Constructs	CE	RTE	SA	SH	SM	SS
Cognitive Engagement (CE)	0.727					
Regulation of Emotion (RE)	0.547	0.741				
Self-Awareness (SA)	0.446	0.342	0.792			
Study Habits (SH)	0.59	0.411	0.427	0.724		
Self-Management (SM)	0.407	0.349	0.416	0.386	0.751	
Social Skills (SS)	0.525	0.457	0.454	0.426	0.36	0.787

**Table 3:** Demographics profile of the participants.

Measure	Items	Frequency (n)	Percentage (%)
Gender	Male	185	54.6
	Female	153	45.4
	Total	338	100.0
Background	Rural	138	40.7
	Urban	200	59.3
	Total	338	100.0
Age	Less than 22	255	75.3
	22–30	83	24.7
	Total	338	100.0
Field of Study	Medical Imaging Ultrasonography	156	46.1
	Nutrition Sciences	78	23.2
	Physiotherapy	104	30.7
	Total	338	100.0

**Table 4:** Collinearity and model fit.

Variables	Cognitive Engagement	Social Skills	Model Fit
Cognitive Engagement		1.737	SRMR: 0.070 NFI: 0.873 RMS_Theta: 0.13
Regulation of Emotion	1.320	1.515	
Self-Awareness	1.440	1.490	
Self-Management	1.309	1.332	
Social Skills	1.486	1.587	

**Table 5:** R square.

Constructs	R Square	R Square Adjusted
Cognitive Engagement	0.440	0.418
Study Habits	0.407	0.399

**Table 6:** Descriptive analysis.

Constructs	N	Minimum	Maximum	*x*	SD
Self-Awareness	338	1.00	7.00	5.235	1.269
Self-Motivation	338	1.00	7.00	4.668	1.304
Regulation of Emotion	338	1.00	7.00	4.650	1.204
Social Skills	338	1.00	7.00	5.288	1.324
Cognitive Engagement	338	1.00	7.00	4.802	1.221
Study Habits	338	1.00	7.00	5.262	1.141

Abbreviations: *x*, mean; SD, standard deviation.

**Table 7:** Direct relationships.

Direct Relationships	Coefficients	*x*	SD	*t*	*p*	Results
SA -> SH	0.121	0.12	0.061	1.98	0.048	Accepted
SM -> SH	0.123	0.124	0.049	2.494	0.013	Accepted
RE -> SH	0.094	0.095	0.053	1.788	0.074	Rejected
SS -> SH	0.078	0.08	0.067	1.165	0.244	Rejected
SA -> CE	0.164	0.164	0.049	3.317	0.001	Accepted
SM -> CE	0.134	0.136	0.051	2.637	0.008	Accepted
RE -> CE	0.330	0.33	0.051	6.472	0.000	Accepted
SS -> CE	0.252	0.253	0.050	4.988	0.000	Accepted
CE -> SH	0.370	0.369	0.065	5.692	0.000	Accepted
Gender -> SH	0.117	0.118	0.046	2.566	0.01	
Field of Study -> SH	−0.068	−0.067	0.049	1.38	0.168	

Abbreviations: SA, self-awareness; SM, self-motivation; RTE, regulate the emotions; CE, cognitive engagement; SH, study habits; *x*, mean; SD, standard deviation.

**Table 8:** Direct relationships.

Direct Relations	Coefficients	*x*	SD	*t*	*p*	Results
SA -> CE -> SH	0.061	0.061	0.021	2.824	0.005	Accepted
SM -> CE -> SH	0.049	0.050	0.021	2.347	0.019	Accepted
RE -> CE -> SH	0.122	0.122	0.030	4.006	0.000	Accepted
SS -> CE -> SH	0.093	0.093	0.025	3.772	0.000	Accepted

Abbreviations: SA, self-awareness; SM, self-motivation; RE, regulation of emotion; CE, cognitive engagement; SH, study habits, *x*, mean; SD, standard deviation.
